# MACSNVdb: a high-quality SNV database for interspecies genetic divergence investigation among macaques

**DOI:** 10.1093/database/baaa027

**Published:** 2020-05-04

**Authors:** Lianming Du, Tao Guo, Qin Liu, Jing Li, Xiuyue Zhang, Jinchuan Xing, Bisong Yue, Jing Li, Zhenxin Fan

**Affiliations:** 1 Institute for Advanced Study, Chengdu University, 2025 Chengluo Rd, Chengdu 610106, China; 2 Department of Obstetrics and Gynecology, West China Second University Hospital, Sichuan University, 20 Section 3, South Renmin Rd, Chengdu 610041, China; 3 Key Laboratory of Bio-Resources and Eco-Environment, Ministry of Education, College of Life Science, Sichuan University, 29 Wangjiang Rd, Chengdu 610065, China; 4 College of Life Sciences and Food Engineering, Yibin University, 8 Wuliangye Rd, Yibin 644000, China; 5 Department of Genetics, Rutgers, the State University of New Jersey, 145 Bevier Rd, Piscataway, NJ 08854, USA

## Abstract

Macaques are the most widely used non-human primates in biomedical research. The genetic divergence between these animal models is responsible for their phenotypic differences in response to certain diseases. However, the macaque single nucleotide polymorphism resources mainly focused on rhesus macaque (*Macaca mulatta*), which hinders the broad research and biomedical application of other macaques. In order to overcome these limitations, we constructed a database named MACSNVdb that focuses on the interspecies genetic diversity among macaque genomes. MACSNVdb is a web-enabled database comprising ~74.51 million high-quality non-redundant single nucleotide variants (SNVs) identified among 20 macaque individuals from six species groups (*muttla*, *fascicularis*, *sinica*, *arctoides*, *silenus*, *sylvanus*). In addition to individual SNVs, MACSNVdb also allows users to browse and retrieve groups of user-defined SNVs. In particular, users can retrieve non-synonymous SNVs that may have deleterious effects on protein structure or function within macaque orthologs of human disease and drug-target genes. Besides position, alleles and flanking sequences, MACSNVdb integrated additional genomic information including SNV annotations and gene functional annotations. MACSNVdb will facilitate biomedical researchers to discover molecular mechanisms of diverse responses to diseases as well as primatologist to perform population genetic studies. We will continue updating MACSNVdb with newly available sequencing data and annotation to keep the resource up to date.

**Database URL**: http://big.cdu.edu.cn/macsnvdb/

## Introduction

Macaques (Cercopithecidae: *Macaca*) are the most widespread non-human primates (1–3), comprising 22 extant species classified into seven species groups, including *sylvanus*, *silenus*, *sinica*, *fascicularis, mulatta, arctoides, sulawesi* (4). Macaques and human shared a last common ancestor about 25 million years ago (5), and their genome sequences share 93.5% identity (6). Due to the genetic and physiological similarity with human, macaques have been widely used in biomedical research, particularly in vaccine and drug development and as animal models for human diseases (7–9). Macaque genes exhibit extremely high sequence similarity with human disease gene orthologs and drug targets (10). However, the interspecies genetic divergence between macaques is believed to explain a large proportion of the observed phenotypic differences in clinical studies. For example, Indian and Chinese rhesus macaques showed significant differences in host response and disease progression after exposed to the same simian immunodeficiency virus (11, 12). Therefore, a comprehensive understanding of the potential variations between these macaque genomes will vastly improve the application of macaques in biomedical research of complex disease.

Single nucleotide variants (SNVs) compose the majority of genetic variations in the macaque genomes and are extensively used as genetic markers for biomedical and evolutionary studies. Non-synonymous SNVs are believed to be largely responsible for the phenotypic variation within populations (13). Moreover, understanding the SNVs locates in macaque disease-related and drug-targeted genes will improve utilization of macaques in the context of biomedical research and provide a valuable resource for pharmacogenomic studies. To date, several databases have been developed to deposit and integrate genotyping data of macaques, such as CMSNP (13), dbSNP (14), MamuSNP (15), MonkeySNP (16), QFbase (17), RhesusBase (18) and mGAP (19). However, these databases are primarily focused on genomic variations within rhesus macaques and the web interfaces of several databases are no longer accessible (Table 1). Additionally, a ban on the export of Indian rhesus macaques has greatly increased the need of other macaques as animal models (20). Therefore, it is imperative to determine the genetic variation among different macaque genomes, which would be crucial for using different macaque species and populations as disease models.

**Table 1:** Comparison of macaque genetic variation database

**Database name**	**Database link**	**Availability**	**Genotype data**	**No. of species**	**Species name or description**
dbSNP	https://www.ncbi.nlm.nih.gov/snp/	Yes	SNPs	2	*Macaca mulatta*, *Macaca fascicularis*
MamuSNP	http://mamusnp.ucdavis.edu	No	SNPs	1	*Macaca mulatta*
MonkeySNP	http://monkeysnp.ohsu.edu/snp/	No	SNPs	2	A mirror of the NCBI dbSNP database
CMSNP	http://monkey.genomics.org.cn/	No	SNPs, SVs	1	*Macaca mulatta*
QFbase	http://genebank.nibio.go.jp/qfbase/	No	cDNA clones	1	*Macaca fascicularis*
RhesusBase	http://rhesusbase.cbi.pku.edu.cn/	Yes	SNPs	1	*Macaca mulatta*
mGAP	https://mgap.ohsu.edu/	Yes	SNVs, Indels	1	*Macaca mulatta*
MACSNVdb	http://big.cdu.edu.cn/macsnvdb/	Yes	SNVs	10	Ten macaque species representing six species groups

SNPs: single nucleotide polymorphism; SNVs: single nucleotide variants; SVs: structural variations

Here, we present a database named MACSNVdb, which provides a user-friendly web interface for accessing and retrieving macaque genome SNV genotyping data. We collected SNVs in 20 individuals from 10 different macaque species, which represent six species groups of the genus *Macaca* (Table 1). The database is designed to display individual SNVs, group- or species-specific SNVs, as well as genotype comparison of non-redundant SNVs (nrSNVs, nucleotides in the reference genome identified as SNV in at last one species) in each sample. MACSNVdb enables users to search synonymous and non-synonymous SNVs within disease-associated or drug-target genes. In addition, we have integrated multiple SNV annotations to enhance the functionality of our database. Finally, we also incorporated functional annotation of genes, including GO term, KEGG pathway, Pfam domain and InterPro, to facilitate users to determine functional impact of SNVs.

## Materials and methods

### Data collection

In this study, we collected 20 macaque resequencing genomes (Table 1) from our studies (20, unpublished) and other related works (10, 17, 21). These macaques covered six species groups of the genus *Macaca*. Six individuals belong to the *mulatta* group (three Chinese rhesus macaques (CR), one Taiwanese macaque (TW), two Japanese macaques (JM), five crab-eating macaques (ce)) belong to the *fascicularis* group, and three individuals belong to the *sinica* group (two Tibetan macaques (TM), and one Assamese macaque (AM)). In addition, three *silenus* group samples (two southern pig-tailed macaques (PM) and one lion-tailed macaque (LM)) and two stump-tailed macaques (SM) belonging to the arctoides group were included. At last, one *sylvanus* group species, Barbary macaque (BM), which is the only species within the *sylvanus* group, was also collected (Table 2).

**Table 2:** Summary of re-sequencing datasets

Group	Species	Common name	Code	Data
*mulatta*	*Macaca mulatta lasiota*	Chinese rhesus macaque	CR1	SRA023856 (NCBI)
*mulatta*	*Macaca mulatta lasiota*	Chinese rhesus macaque	CR2	SRA037810 (NCBI)
*mulatta*	*Macaca mulatta lasiota*	Chinese rhesus macaque	CR3	PRJNA239805 (NCBI)
*mulatta*	*Macaca cyclopis*	Taiwanese macaque	TW1	N/A
*mulatta*	*Macaca fuscata fuscata*	Japanese macaque	JM1	DRP000620 (NCBI)
*mulatta*	*Macaca fuscata fuscata*	Japanese macaque	JM2	N/A
*fascicularis*	*Macaca fascicularis*	Crab-eating macaque	CE1	SRA023855 (NCBI)
*fascicularis*	*Macaca fascicularis*	Crab-eating macaque	CE2	DRA000430 (DDBJ)
*fascicularis*	*Macaca fascicularis*	Crab-eating macaque	CE3	PRJNA20409 (NCBI)
*fascicularis*	*Macaca fascicularis*	Mauritian crab-eating macaque	CE4	PRJEB7871 (EMBL-EBI)
*fascicularis*	*Macaca fascicularis*	Mauritian crab-eating macaque	CE5	PRJEB7871 (EMBL-EBI)
*sinica*	*Macaca thibetana*	Tibetan macaque	TM1	SRP032525 (NCBI)
*sinica*	*Macaca thibetana*	Tibetan macaque	TM2	N/A
*sinica*	*Macaca assamensis*	Assamese Macaque	AM1	PRJNA305009 (NCBI)
*arctoides*	*Macaca arctoides*	Stump-tailed macaque	SM1	PRJNA305009 (NCBI)
*arctoides*	*Macaca arctoides*	Stump-tailed macaque	SM2	PRJNA305009 (NCBI)
*silenus*	*Macaca nemestrina*	Southern pig-tailed macaque	PM1	SRS693104 (NCBI)
*silenus*	*Macaca nemestrina*	Southern pig-tailed macaque	PM2	SRS693108 (NCBI)
*silenus*	*Macaca silenus*	Lion-tailed macaque	LM1	N/A
*sylvanus*	*Macaca sylvanus*	Barbary macaque	BM1	N/A

N/A indicates that the sequencing data was not published and can be downloaded from our server.

### Data processing

The paired-end short reads were aligned to the Indian rhesus macaque genome (rheMac8) using Bowtie2 (22) under the local alignment algorithm with the very sensitive model and proper insert sizes. Default options were used for other parameters. Next, Picard (http://broadinstitute.github.io/picard) and GATK toolsets (23) were applied to process the alignments to SNV calls. The whole pipeline converted the short reads to bam format alignment files, and then generated genotype calls in Variant Call Format (VCF). The pipeline is the same as used in our previous studies (20, 24–26). Based on the raw SNV calls, a series of data quality filters were applied to improve the quality of genotype calls. These filters were grouped into two levels: genome filters, which was based on the reference genome’s features and polymorphism across all the samples, and sample filters, which was based on the genotype calls of each sample. The genome filters contained the following parameters: (i) triallelic sites would be filtered out due to their higher genotyping errors; (ii) mutations within copy number variant (CNV) regions would be filtered out due to higher false-positive rates within CNVs. The sample filters contained the following criteria: (i) SNVs near indels (5 bp, either up or downstream) were excluded; (ii) SNVs within 5 bp of another SNV were excluded; (iii) minimum genotype quality threshold of 20 (*P*[error] = 0.01) for each mutation; (iv) minimum genotype quality threshold of 30 (*P*[error] = 0.001) for each mutation.

The macaque orthologs of human genes were extracted from HCOP (27) and only one-to-one orthologs were used to link macaque genes with disease genes downloaded from the OMIM database (last accessed May 8, 2019) (28) and drug-targeted genes obtained from DrugBank database v5.1.4 (29). All nrSNVs in the coding region were then annotated and assigned to macaque orthologs of human disease genes and drug-targeted genes. The potential group-specific and species-specific SNVs were determined using the following criteria. The group-specific SNVs must be homozygous in all samples within the group, present in only one group, and non-variant in other groups. Similarly, the species-specific SNVs must be homozygous in all samples within the species, present in only one species, and non-variant in other species.

### Database implementation

**Figure 1:**
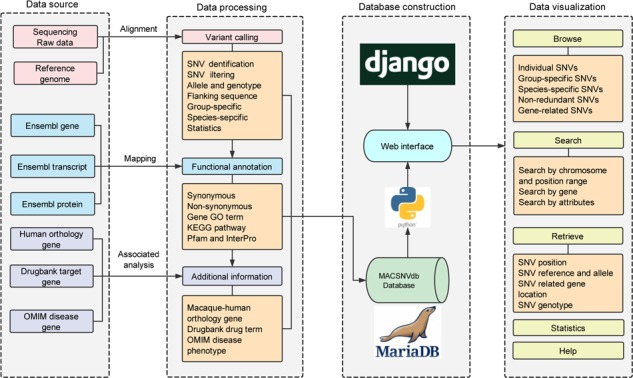
Database construction overview and the functionality of MACSNVdb.

Several summarizing tables including high-quality SNVs and their annotations were precomputed with Python scripts (https://github.com/lmdu/macaca). All the metadata were organized and stored in a MariaDB relational database (https://mariadb.org/) operated on a CentOS server. The interactive web interface has been built with Python and the Django web framework (https://www.djangoproject.com/). The web pages were constructed using HTML5, CSS3 and rendered using Jinja2 (http://jinja.pocoo.org/) template engine. Jquery library was used with Semantic UI framework (https://semantic-ui.com/) to establish a responsive user-friendly frontend interface. Construction process and features of the database is schematically illustrated in Figure 1.

**Figure 2:**
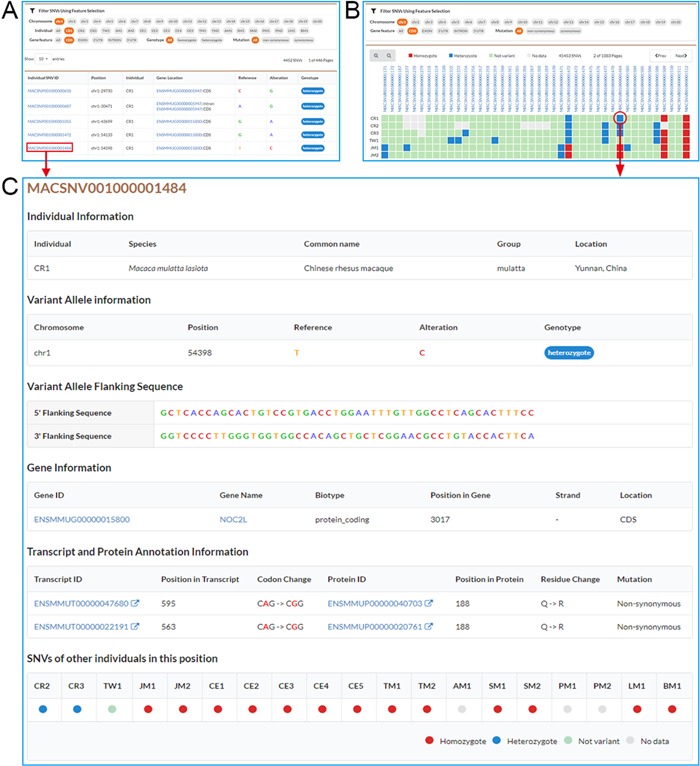
Screenshots of SNV browse pages. **A**. Individual SNVs’ browse page contains the filtration pane with the SNV result table below. By using the filtration pane, users can filter SNV data and get a list of relevant SNVs. **B**. Non-redundant SNVs’ browse page. Comparative map of genotypes in each individual is indicated by colored and filled circles in SNV result table. **C**. Detailed information for an SNV.

## Results

### Database content and statistics

In total, 193.13 million SNVs were detected from 20 macaque genomes, of which 116.81 million were homozygous and 76.32 million were heterozygous, represent 74.51 million nrSNVs, with a density of 28.24 SNVs/kb and an average length of SNV intervals of 33.5 bp. We mapped all nrSNVs to annotated macaque genes and found that 45.75 million (61.41%) nrSNVs were present in intergenic region, while 28.75 million (38.59%) nrSNVs were present in genic region. Among genic nrSNVs, about 96.67% fell into intron regions whereas only 486 697 (1.69%) nrSNVs lay in coding regions, of which 243 147 nrSNVs were non-synonymous, accounting for about 49.96% of nrSNVs in the coding regions.

In order to enable an easy application of our database in biomedical research, we have connected nrSNVs in the coding region to human diseases and drug targets through macaque orthologs of human genes. In aggregate, we extracted 2083 macaque orthologs of human disease genes, of which 2003 orthologs contained at least one nrSNV in coding regions, and 1941 orthologs contained at least one non-synonymous nrSNV. In all, we detected 75 931 nrSNVs within the coding region of these genes, of which 47 662 were synonymous and 28 739 were non-synonymous. Additionally, we also obtained 1452 macaque orthologs of human drug-targeted genes. Among them, 1415 orthologs possessed at least one nrSNV in the coding region, and 1359 orthologs had at least one non-synonymous nrSNVs. Overall, we identified 40 910 nrSNVs within the coding regions of these genes, of which 14 500 were non-synonymous and 26 690 were synonymous.

The 20 macaque individuals represent six species groups and 10 species. Accordingly, we observed a total of 5.22 million potential group-specific SNVs and 6.77 million potential species-specific SNVs. The majority of group-specific SNVs were present in the *sylvanus* group, accounting for 80.27% of all group-specific SNVs, followed by the *arctoides* group (9.41%), the *silenus* group (6.63%) and the *sinica* group (3.68%), while only 372 SNVs were specific to the *mulatta* group. These significant differences in group-specific SNVs might result from the phylogenetic relationships among them. Our reference genome was *M. mulatta*, which belonged to the *mulatta* group, while the *sylvanus* group has the furthest genetic distance to the *mulatta* group. Among 10 species, *M. sylvanus* possessed the largest amount of species-specific SNVs, accounting for 61.95%. *M. mulatta lasiota* had with 2123 SNVs the fewest specific SNVs.

### Web interface and usage

The MACSNVdb database provides a user-friendly web interface that facilitates users to browse, search and retrieve macaque genome SNVs. Six functional units in the top navigation bar including “Home”, “Browse”, “Genes”, “Statistics”, “Retrieve” and “Help” were designed to assist users to access the database. The “Home” page offers a quick search function for immediate redirection to a query item by typing a SNV unique identifier, an OMIM phenotype entry number or a drug accession number from DrugBank.

Users may browse individual, non-redundant and specific SNVs by using submenus under “Browse” function unit. In the “Browse” page, users can use the flexible filtering functionality in the filtration pane to filter SNVs by chromosome, individual, gene features (CDS, UTR, In-tron), genotype (homozygote, heterozygote) or mutation type (non-synonymous, synonymous), and a list of matched SNVs is returned in the result table under the filtration pane (Figure 2A). MACSNVdb also allows users to investigate the comparison of variations between individuals by using filled circles with different colors to indicate genotypes (Figure 2B). By clicking the SNV identifier hyperlink in the SNV result table, users can obtain detailed SNV information (Figure 2C). Basic information includes individual information, chromosome position, reference and altered alleles, and 5′ and 3′ flanking sequences. Additional information includes annotated gene, transcript and protein, altered codon and amino acid residue, mutation type and genotypes from other individuals. Moreover, users may acquire further gene functional annotations such as GO terms, KEGG pathways, Pfam domains and InterPro domains by clicking the gene identifier hyperlink. We also provide an advanced table to sort and search disease or drug-target genes and help users to easily retrieve SNVs in CDS region of genes for further study.

**Figure 3:**
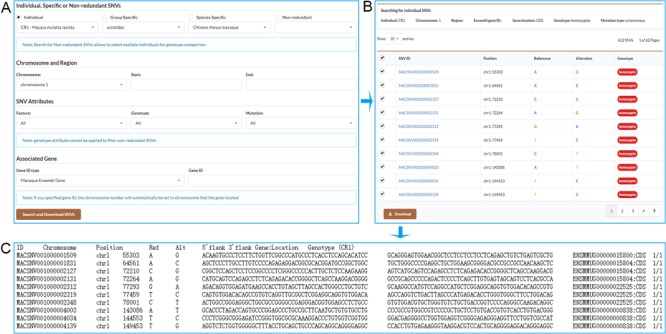
Screenshots of the search result pages. **A**. Search for individual SNVs, specific SNVs or non-redundant SNVs by specifying chromosome and position range or associated gene. **B**. Search result table allows users to select desired SNVs to download. **C**. SNV list with detailed information obtained by downloading.

We provide a robust function for users to retrieve a set of individual, specific or non-redundant SNVs that meet user-specified criteria (Figure 3A). These SNVs can be retrieved by selecting the chromosome and specifying start and end position on the chromosome. Users may also retrieve gene-associated SNVs by inputting an Ensembl gene ID, a drug ID from Drugbank database or an accession number of a phenotype from OMIM database. Users can further select gene feature, genotype and mutation type to restrict the output results. Users may select the desired SNVs to download (Figure 3B), and a tab-separated values file containing SNV detailed information will be returned (Figure 3C).

## Discussion

With human-comparable genomes and many advantages as model animals, macaque species pose a unique model in molecular and translational study of human diseases. Currently, various macaque species such as rhesus macaque, crab-eating macaque, stump-tailed macaque, Tibetan macaque and Japanese macaque have been widely employed to researches related to human diseases or medicine evaluations. Despite the close evolutionary relationships among macaque species, they vary greatly in morphology, physiology and genetics (30, 31). However, few studies paid attentions to the genetic differences of different model animals they adopt, which might lead to inconsistent results among researches. For example, different species (subspecies) of macaque react differently and show different levels of pathogenesis to human infectious diseases such as AIDS (11) and malaria (32). Therefore, it is highly desirable to provide user-friendly tools to present the genetic differences of the different macaque species. MACSNVdb is the most extensive resource focusing on the genetic divergence between different macaque genomes. At present, SNVs within MACSNVdb were collected from 20 individuals of 10 species covering most of the commonly used macaque species in biomedical research. Apart from individual and non-redundant SNVs, MACSNVdb allows users to browse group-specific and species-specific SNVs that might help to explain differences among macaques regarding immune responses and disease progression. We further provided a flexible filtering functionality for screening SNVs of interest, such as non-synonymous SNVs. Another feature of our database is the ability to inquire SNVs within disease-associated or drug-target genes. For each macaque SNV, MACSNVdb also provides the disease-associated or drug-target genes in humans, which could be useful to study the pharmacology and drug response in macaques.

We will continue to expand our database with new publicly available datasets and private data generated by our own research groups. We will continue to make improvements to the SNV mining and annotation algorithms to obtain more accurate SNV data and update the database. We will add analytical functionalities so that users can easily use SNVs-associated data to perform downstream analyses such as enrichment analysis, genetic statistical analysis and phylogenetic analysis. In the meantime, we will improve or adjust the web interface according to users’ feedback to maximize the utilization of the database in biomedical research. Furthermore, we plan to integrate more human disease related information from other databases, such as, DisGeNET (33), MalaCards (34) and ClinVar (35) in the future.

## Conclusion

In conclusion, MACSNVdb not only provides useful information for macaque SNVs but also presents a comparative map of genotypes between macaques. It is anticipated that MACSNVdb will be a useful repository for deciphering the phenotypic differences between macaques in biomedical research and aid in selection of macaques for use as disease models.
